# Comparison of the efficacy between modified prone split-leg position and lithotomy position in retrograde intrarenal surgery for upper urinary tract calculi

**DOI:** 10.3389/fsurg.2026.1959157

**Published:** 2026-10-20

**Authors:** Hongfei Li, Chao Li, Jiahang Liu, Xudong Yang, Yanjun Lin, Shengjie Yu

**Affiliations:** 1 Department of Urology, The Second Affiliated Hospital of Chongqing Medical University, Chongqing, China; 2 Department of Urology, Chamdo People’s Hospital, Qamdo, Tibet, China

**Keywords:** lithotomy position, modified prone split-leg position, propensity score matching, retrograde intrarenal surgery, upper urinary tract calculi

## Abstract

**Purpose:**

To compare the efficacy and safety of the modified prone split-leg position versus the lithotomy position in retrograde intrarenal surgery (RIRS) for upper urinary tract calculi.

**Methods:**

We retrospectively analyzed 270 patients undergoing RIRS for upper urinary tract calculi (November 2024–March 2026): modified prone split-leg (*n* = 103) and lithotomy (*n* = 167). After 1:1 propensity score matching (84 per group), operative time, hospital stay, day-1 stone-free rate (KUB), fever, renal function, and inflammatory markers were compared; subgroup, multivariable regression, and interaction analyses were performed.

**Results:**

After matching, baseline characteristics were balanced. The immediate radiographic stone-free rate (SFR), assessed by KUB on postoperative day 1, was 94.05% vs. 85.71% (*P* = 0.073). Operative time was longer with the modified prone split-leg position (54.00 vs. 38.50 min, *P* < 0.001), as was hospital stay (*P* = 0.012); the fever rate was 1.19% in both groups. In the 10–20 mm subgroup, SFR was significantly higher in the modified prone split-leg group (95.12% vs. 78.05%, *P* = 0.023). The renal stone subgroup showed a numerically higher SFR (92.31% vs. 42.86%, *P* = 0.031) but was hypothesis-generating owing to the small sample size. No SFR difference was found for ureteral stones (*P* = 0.290), and renal function and inflammatory markers did not differ between groups. Multivariable regression showed that the lithotomy position was associated with a higher risk of residual stones (OR = 5.327, *P* = 0.013) and shorter operative time (B = −10.295, *P* = 0.009). Interaction analyses were not significant (*P* for interaction: stone size 0.999, BMI 0.946, stone location 0.059).

**Conclusion:**

In the unadjusted analysis, the immediate radiographic SFR did not differ significantly between the two positions. After adjustment for residual confounders, the modified prone split-leg position was independently associated with a lower risk of residual stones. It was also associated with a higher SFR for 10–20 mm stones; however, the interaction was not significant, and this finding should be interpreted cautiously. In the renal stone subgroup, a numerically higher SFR was observed, but this hypothesis-generating finding requires validation in larger studies.

## 1 Introduction

Upper urinary tract calculi are among the most common diseases in urology in China, and epidemiological data show a continuously increasing prevalence. According to a systematic review and meta-analysis covering 22 provinces in China, the overall prevalence of urolithiasis in the country is approximately 8.1% (95% CI 5.6–11.1%), with renal stones accounting for 7.8% and ureteral stones for 3.2% (1). Retrograde intrarenal surgery (RIRS), which uses a flexible ureteroscope to disintegrate stones via the natural orifice, offers advantages such as minimal trauma, fast recovery, and reliable efficacy. Currently, RIRS is recommended by the International Alliance of Urolithiasis (IAU) guidelines as a first-line treatment for intrarenal or proximal ureteral stones <20 mm in diameter (evidence level 1, recommendation grade A) (2). Compared with extracorporeal shock wave lithotripsy, RIRS has a higher single-session success rate and a lower retreatment rate, thus becoming an important minimally invasive treatment for upper urinary tract calculi (3).

The choice of patient position during RIRS has potential effects on surgical convenience, stone-free rate, and complication rate (4). The conventional lithotomy position is the most commonly used position for RIRS because it is operator-friendly, but it has disadvantages such as a tendency to increase intrarenal pressure and limited visualization of lower-calyceal stones (5, 6). To improve the management of lower-calyceal stones, some surgeons have attempted to adopt the prone position, using gravity to improve lower-calyceal visualization, reduce intrarenal pressure, and increase the stone-free rate (7). However, the prone position also has problems such as time-consuming positioning, poor patient tolerance, and complicated anaesthesia management. In recent years, the modified prone split-leg position, which combines the stone-clearing advantages of the prone position with the operative convenience of the lithotomy position, has gradually attracted attention (8), but its clinical value in RIRS still lacks high-quality evidence.

Currently, comparative studies on the modified prone split-leg position versus the lithotomy position in RIRS are scarce, and most have limitations such as small sample sizes, baseline imbalances, and uncontrolled confounding factors. Existing studies mainly focus on the use of the modified prone split-leg position in combined endoscopic surgery for urinary tract stones, and its evaluation in RIRS alone is insufficient (9). Moreover, whether stone size, BMI, or stone location affects the treatment outcomes of different positions remains unclear (10). Therefore, it is necessary to systematically compare the clinical outcomes of the two positions under conditions that control for confounding bias and to explore the populations that may benefit.

Based on this, the present study retrospectively analyzed the clinical data of 270 patients with upper urinary tract calculi who underwent RIRS at the Second Affiliated Hospital of Chongqing Medical University from November 2024 to March 2026. Propensity score matching (PSM) was used to control baseline bias (11). The differences in postoperative stone-free rate, operative time, hospital stay, complications, and renal function parameters between the modified prone split-leg position and the lithotomy position were compared, and subgroup analyses were performed according to maximum stone diameter, BMI, and stone location. Multivariable logistic regression and multiple linear regression were further performed to control for residual confounding and to identify factors associated with residual stones and operative time, and interaction analyses were conducted to assess whether the effect of surgical position differed across subgroups. The aim was to evaluate the feasibility, safety, and efficacy of RIRS in the modified prone split-leg position and to provide a reference for clinical positioning decisions.

## 2 Methods

### 2.1 Study subjects

A retrospective analysis was conducted on patients with ureteral or renal stones admitted to the Department of Urology, the Yuzhong and Jiangnan campuses of The Second Affiliated Hospital of Chongqing Medical University, from November 2024 to March 2026. All procedures were performed by the same experienced endourology surgical team. The surgical position was not randomly assigned. The choice of position was based on the routine practice at each campus: patients treated at the Yuzhong campus received the modified prone split-leg position, whereas patients treated at the Jiangnan campus received the conventional lithotomy position, reflecting the routine practice at each campus. According to the position actually used, patients were retrospectively assigned to an observation group (modified prone split-leg position) and a control group (lithotomy position). Propensity score matching was used to balance baseline characteristics between the two groups. Following application of the inclusion and exclusion criteria described below, a total of 270 patients were finally enrolled, including 103 in the modified prone split-leg group and 167 in the lithotomy group. Due to baseline imbalances between the two groups, 1:1 propensity score matching (PSM) was performed, and 168 patients (84 pairs) were included for analysis after matching.

Inclusion criteria: (a) patients with a single ureteral or renal stone clearly diagnosed by imaging, with or without symptoms; (b) complete clinical data and medical records without missing information.

Exclusion criteria: (a) concurrent bilateral urinary stone surgery; (b) severe liver dysfunction or coagulation abnormalities; (c) congenital ureteral stricture, etc.

This study complied with the Declaration of Helsinki and was approved by the Medical Ethics Committee of the Second Affiliated Hospital of Chongqing Medical University. Due to the retrospective nature of the study using anonymized clinical data only, the requirement for written informed consent was waived. All patients had provided written informed consent for the operation prior to surgery.

### 2.2 Surgical procedure

Patients in the control group were placed in the conventional lithotomy position. Patients in the observation group were placed in the modified prone split-leg position: the non-operative side lower limb was abducted to 90° with the hip and knee flexed and abducted to approximately 90° and placed on a leg support, while the operative side lower limb remained in line with the leg support and was abducted to approximately 15°. Surgical procedure: after tracheal intubation under general anesthesia, routine disinfection and draping were performed. A rigid ureteroscope was inserted transurethrally to perform cystoscopy and examine the ipsilateral ureteral orifice. A Zebra guidewire was inserted retrogradely into the ureter under direct vision, and a ureteral access sheath (UAS) was advanced over the guidewire. A disposable Zebra electronic flexible ureteroscope was inserted through the sheath to accurately locate the stone. Holmium laser lithotripsy was performed (left pedal: 20 Hz, 1.0 J; right pedal: 45 Hz, 2.0 J) to pulverize the stone to ≤2 mm fragments. Larger fragments were retrieved with a stone basket. A ureteral stent was routinely placed after surgery.

### 2.3 Observation indicators and evaluation criteria

#### 2.3.1 Operative indicators

Operative time and postoperative hospital stay were recorded. Operative time was defined as the endoscopic procedure time, measured from the insertion of the ureteroscope to the removal of the ureteral access sheath. This definition did not include patient positioning, anesthesia induction, or recovery time; therefore, it reflects the intracorporeal manipulation time rather than total room time (12); postoperative hospital stay was defined as the number of days from surgery to discharge (13).

#### 2.3.2 Postoperative complications

Postoperative fever was defined as axillary temperature ≥38.0 °C within 72 h after surgery (14). This criterion was used as a preliminary indicator for comparing short-term safety outcomes between the two positions, acknowledging that it does not capture the full spectrum of perioperative complications.

#### 2.3.3 Immediate radiographic stone-free rate

The immediate radiographic stone-free rate (SFR) was assessed by plain abdominal x-ray (KUB) on postoperative day 1. According to domestic and international consensus, stone clearance was defined as no visible residual stone or a residual stone maximum diameter <4 mm (2, 15). Some patients underwent subsequent CT evaluation based on clinical needs; however, because CT was not performed in all patients, these data were not systematically analyzed and were not included in the SFR assessment.

#### 2.3.4 Laboratory indicators

Approximately 3 mL of fasting venous blood was collected from each patient preoperatively (before surgery after admission) and on postoperative day 1. Blood samples were placed into anticoagulant tubes containing disodium ethylenediaminetetraacetic acid (EDTA-2Na), centrifuged at 5,000 r/min for 10 min (centrifugal radius 8 cm), and the plasma was separated for later use. Plasma creatinine (Scr) and plasma urea nitrogen (BUN) levels were measured using an automatic biochemical analyzer (16); white blood cell (WBC) count was measured using a hematology analyzer (17); C-reactive protein (CRP) level was measured by enzyme-linked immunosorbent assay (ELISA). The changes in each indicator (postoperative value minus preoperative value) were also calculated.

### 2.4 Statistical methods

Statistical analyses were performed using SPSS 26.0 and R 4.3.0 software. A *P* value <0.05 was considered statistically significant.

Continuous variables were first tested for normal distribution using the Shapiro-Wilk test. Normally distributed variables are presented as mean ± standard deviation, and between-group comparisons were performed using the independent samples t-test. Non-normally distributed variables are presented as median (interquartile range, P₂₅, P₇₅), and between-group comparisons were performed using the Mann-Whitney U test. Categorical variables are presented as frequencies (percentages), and between-group comparisons were performed using the Pearson *χ*² test, with Fisher’s exact test used when the expected frequency was <5.

To control for baseline differences between the two groups (age, mean stone CT value, and preoperative ureteral stent placement rate, all *P* < 0.05 before PSM), 1:1 nearest-neighbor propensity score matching (PSM) was performed using SPSS 26.0. The surgical position was used as the dependent variable; age, mean stone CT value, and preoperative ureteral stent placement rate were used as covariates. The caliper value was set to 0.02, sampling was performed without replacement, and the random seed was set to 42. After matching, standardized mean difference (SMD) was calculated to evaluate covariate balance, and SMD <0.25 indicated acceptable balance between the two groups. This threshold was chosen *a priori* based on the propensity score methodology literature, in which SMD values below 0.25 have been considered indicative of acceptable balance (18, 19), and reflects a deliberate trade-off: stricter calipers or additional matching covariates would have further reduced the number of matched pairs and, consequently, the already limited number of residual-stone events available for multivariable modeling. Between-group comparisons after matching were performed using the same methods as before matching. Subgroup analyses (stratified by maximum stone diameter, BMI, and stone location) were conducted using the same comparative methods. No adjustment for multiple comparisons was performed, and subgroup analyses were considered exploratory.

Multivariable logistic regression was performed to identify factors associated with postoperative residual stones, and multiple linear regression was used to identify factors associated with operative time and postoperative hospital stay. Variables included in the models were surgical position, maximum stone diameter, preoperative urine culture, and stone location. Interaction analyses were performed by including a “surgical position × subgroup” interaction term in the logistic model, and the Wald test was used to obtain the *P for interaction*.

## 3 Results

### 3.1 General data analysis

A total of 270 patients with urinary calculi who underwent RIRS were enrolled in this study, including 103 patients in the observation group (modified prone split-leg position) and 167 patients in the control group (lithotomy position). Before PSM, there were no statistically significant differences between the two groups in terms of sex composition (male: 66.02% vs. 68.86%, *P* = 0.627), BMI (24.80 vs. 24.40 kg/m², *P* = 0.544), prevalence of hypertension (37.86% vs. 34.13%, *P* = 0.534), prevalence of diabetes (20.39% vs. 14.37%, *P* = 0.198), maximum stone diameter (10.00 vs. 10.00 mm, *P* = 0.418), stone laterality (*P* = 0.305), stone location (*P* = 0.794), preoperative renal function indicators (plasma creatinine, plasma urea nitrogen), or inflammatory markers (C-reactive protein, white blood cell count). Compared with the control group, the observation group had a higher age (58 years vs. 53 years, *P* < 0.001), a lower mean stone CT value (883.54 HU vs. 1,000.55 HU, *P* < 0.001), and a higher rate of preoperative ureteral stent placement (40.78% vs. 25.15%, *P* = 0.007). The difference in preoperative urine culture positivity rate between the two groups was of borderline statistical significance (44.66% vs. 33.53%, *P* = 0.067) (Table 1).

**Table 1:** Comparison of clinical data between the two groups before PSM.

Variable	Observation group (*n* = 103)	Control group (*n* = 167)	t/X^2^/Z	*P*
Sex, *n* (%)			0.236	0.627
Male	68 (66.02%)	115 (68.86%)		
Female	35 (33.98%)	52 (31.14%)		
Age (years), median (P25, P75)	58 (51,65)	53 (42,59)	−3.693	＜0.001
Hypertension, *n* (%)	39 (37.86%)	57 (34.13%)	0.387	0.534
Diabetes, *n* (%)	21 (20.39%)	24 (14.37%)	1.661	0.198
BMI (kg/m²), median (P25, P75)	24.80 (22.00,26.30)	24.40 (22.90,26.50)	−0.607	0.544
Preoperative urine culture positive, *n* (%)	46 (44.66%)	56 (33.53%)	3.356	0.067
Preoperative ureteral stent placement, *n* (%)	42 (40.78%)	42 (25.15%)	7.259	0.007
Maximum stone diameter (mm), median (P25, P75)	10.00 (8.00,15.00)	10.00 (8.00,13.00)	−0.810	0.418
Stone laterality, *n* (%)			1.052	0.305
Left	51 (49.51%)	72 (43.11%)		
Right	52 (50.49%)	95 (56.89%)		
Stone location, *n* (%)			0.068	0.794
Kidney	16 (15.53%)	24 (14.37%)		
Ureter	87 (84.47%)	143 (85.63%)		
Mean stone CT value (HU), mean ± SD	883.54 ± 244.09	1000.55 ± 236.51	−3.819	＜0.001
Preoperative plasma creatinine (*μ*mol/L), median (P25, P75)	77.60 (67.30,97.00)	77.20 (62.00,96.90)	−1.065	0.287
Preoperative plasma urea nitrogen (mmol/L), median (P25, P75)	5.90 (4.76,7.20)	5.52 (4.66,6.58)	−1.474	0.141
Preoperative C-reactive protein (mg/L), median (P25, P75)	5.00 (5.00,6.89)	5.00 (5.00,6.44)	−0.403	0.687
Preoperative white blood cell count (×10⁹/L), median (P25, P75)	6.60 (5.09,8.17)	6.51(5.41,7.88)	−0.053	0.958

### 3.2 Comparison of baseline data between the two groups after propensity score matching

To eliminate confounding bias, 1:1 propensity score matching (PSM) was performed, and 84 patients were included in each group. After matching, there were no statistically significant differences between the two groups in terms of sex (male: 70.24% vs. 64.29%, *P* = 0.411), age (55.68 ± 11.02 vs. 56.43 ± 11.69 years, *P* = 0.669), BMI (24.42 ± 2.95 vs. 24.71 ± 3.38 kg/m², *P* = 0.550), prevalence of hypertension (36.90% vs. 38.10%, *P* = 0.873), prevalence of diabetes (17.86% vs. 14.29%, *P* = 0.529), preoperative urine culture positivity rate (45.24% vs. 30.95%, *P* = 0.057), preoperative ureteral stent placement rate (33.33% vs. 32.14%, *P* = 0.869), maximum stone diameter (11.00 vs. 10.00 mm, *P* = 0.133), stone laterality (*P* = 0.217), stone location (*P* = 0.153), mean stone CT value (899.89 vs. 920.89 HU, *P* = 0.574), as well as preoperative renal function indicators and inflammatory markers (all *P* > 0.05), indicating that the baseline characteristics of the two groups were well balanced and comparable.

After PSM, most covariates were well balanced (SMD < 0.25; the full set of post-matching SMDs is presented in Table 2). The SMDs for maximum stone diameter (0.317) and preoperative urine culture positivity (0.297) slightly exceeded 0.25; these two variables, together with stone location (included *a priori* for its clinical relevance), were entered as covariates in the multivariable logistic and linear regression models to further control for their potential confounding effects.

**Table 2:** Comparison of clinical data between the two groups after PSM.

Variable	Observation group (*n* = 84)	Control group (*n* = 84)	t/X^2^/Z	*P*	SMD
Sex, *n* (%)			0.676	0.411	0.127
Male	59 (70.24%)	54 (64.29%)			
Female	25 (29.76%)	30 (35.71%)			
Age (years), mean ± SD	55.68 ± 11.02	56.43 ± 11.69	−0.428	0.669	−0.066
Hypertension, *n* (%)	31 (36.90%)	32 (38.10%)	0.025	0.873	−0.025
Diabetes, *n* (%)	15 (17.86%)	12 (14.29%)	0.397	0.529	0.097
BMI (kg/m²), mean ± SD	24.42 ± 2.95	24.71 ± 3.38	−0.600	0.550	−0.093
Preoperative urine culture positive, *n* (%)	38 (45.24%)	26 (30.95%)	3.635	0.057	0.297
Preoperative ureteral stent placement, *n* (%)	28 (33.33%)	27 (32.14%)	0.027	0.869	0.025
Maximum stone diameter (mm), median (P25, P75)	11.00 (8.03,16.15)	10.00 (8.13,12.70)	−1.501	0.133	0.317
Stone laterality, *n* (%)			1.527	0.217	0.191
Left	44 (52.38%)	36 (42.86%)			
Right	40 (47.62%)	48 (57.14%)			
Stone location, *n* (%)			2.043	0.153	0.222
Kidney	13 (15.48%)	7 (8.33%)			
Ureter	71 (84.52%)	77 (91.67%)			
Mean stone CT value (HU), mean ± SD	899.89 ± 243.18	920.89 ± 240.06	−0.563	0.574	−0.087
Preoperative plasma creatinine (μmol/L), median (P25, P75)	78.10 (67.63,97.83)	77.25 (61.30,98.65)	−0.787	0.431	0.081
Preoperative plasma urea nitrogen (mmol/L), median (P25, P75)	5.90 (4.98,7.22)	5.76 (4.68,6.90)	−0.871	0.384	0.081
Preoperative C-reactive protein (mg/L), median (P25, P75)	5.00 (5.00,5.15)	5.00 (5.00,6.68)	−1.203	0.229	−0.095
Preoperative white blood cell count (×10^9^/L), median (P25, P75)	6.38 (5.03,7.87)	6.14 (5.16,7.59)	−0.493	0.622	0.134

### 3.3 Comparison of major surgical outcome indicators

After PSM, comparison of surgical outcome indicators between the two groups showed that the immediate radiographic stone-free rate (SFR), defined as no residual stone ≥4 mm on postoperative day 1 KUB, was 94.05% in the observation group (modified prone split-leg position) and 85.71% in the control group (lithotomy position), with no statistically significant difference (*P* = 0.073). The postoperative fever rate was 1.19% in both groups (*P* = 1.000). Operative time was significantly longer in the observation group [54.00 (35.00, 70.00) min] than in the control group [38.50 (29.00, 50.00) min] (*P* < 0.001). Postoperative hospital stay was also longer in the observation group [1 (1, 2) d] than in the control group [1 (1, 1) d] (*P* = 0.012). The difference in postoperative plasma creatinine level between the two groups was of borderline statistical significance (*P* = 0.063), and postoperative plasma urea nitrogen was higher in the observation group than in the control group (*P* = 0.013). There were no statistically significant differences between the two groups in postoperative C-reactive protein (*P* = 0.862) or white blood cell count (*P* = 0.920). The changes in plasma creatinine (*P* = 0.063), plasma urea nitrogen (*P* = 0.136), C-reactive protein (*P* = 0.957), and white blood cell count (*P* = 0.503) also showed no statistically significant differences between the two groups (Table 3).

**Table 3:** Comparison of postoperative outcomes between the two groups after PSM.

Variable	Observation group (*n* = 84)	Control group (*n* = 84)	t/X^2^/Z	*P*
Postoperative day 1 SFR, *n* (%)	79 (94.05%)	72 (85.71%)	3.207	0.073
Postoperative fever, *n* (%)	1 (1.19%)	1 (1.19%)	0.000	1.000
Operative time (min), median (P25, P75)	54.00 (35.00,70.00)	38.50 (29.00,50.00)	−3.816	＜0.001
Postoperative hospital stay (d), median (P25, P75)	1 (1,2)	1 (1,1)	−2.502	0.012
Postoperative plasma creatinine (μmol/L), median (P25, P75)	81.80 (66.58,92.58)	71.85 (61.45,92.10)	−1.856	0.063
Postoperative plasma urea nitrogen (mmol/L), median (P25, P75)	4.98 (4.37,6.56)	4.53 (3.79,5.92)	−2.482	0.013
Postoperative C-reactive protein (mg/L), median (P25, P75)	5.00 (5.00,6.32)	5.00 (5.00,7.02)	−0.174	0.862
Postoperative white blood cell count (×10⁹/L), median (P25, P75)	7.37 (5.90,9.39)	7.59 (5.98,9.33)	−0.100	0.920
Change in plasma creatinine (μmol/L), median (P25, P75)	0.30(−6.63,6.70)	−2.80(−8.58,3.38)	−1.861	0.063
Change in plasma urea nitrogen (mmol/L), mean ± SD	−0.85 ± 1.40	−1.17 ± 1.36	−1.497	0.136
Change in C-reactive protein (mg/L), median (P25, P75)	0.00 (0.00,0.00)	0.00 (0.00,0.00)	−0.054	0.957
Change in white blood cell count ( × 10⁹/L), median (P25, P75)	0.81(−0.48,2.50)	1.00(−0.48,2.71)	−0.669	0.503

To further control for residual confounding after propensity score matching, multivariable logistic regression and multiple linear regression analyses were performed, adjusting for maximum stone diameter, preoperative urine culture, and stone location (Tables 4, 5).

**Table 4:** Binary logistic regression analysis of factors associated with postoperative residual stones (*n* = 168).

Variable	B	Wald X^2^	*p*	OR	95%CI
Surgical position	1.673	6.188	0.013	5.327	(1.426～19.904)
Maximum stone diameter (mm)	0.165	9.510	0.002	1.179	(1.062～1.310)
Preoperative urine culture (positive)	−0.136	0.054	0.816	0.873	(0.276～2.759)
Stone location (kidney vs. ureter)	0.773	1.206	0.272	2.165	(0.545～8.595)

Reference group, modified prone split-leg position; dependent variable, residual stones on postoperative day 1 x-ray (1 = residual, 0 = clearance); OR, odds ratio; CI, confidence interval. In a sensitivity analysis using Firth’s penalized logistic regression, the lithotomy position remained associated with residual stones (B = 1.525, SE = 0.631, OR = 4.595, 95% CI 1.335–15.819, *P* = 0.016).

**Table 5:** Multiple linear regression analysis of operative time and postoperative hospital stay (*n* = 168).

Variable	Operative time (min)		Postoperative hospital stay (d)	
	B (95%CI)	P	B (95%CI)	P
Surgical position	−10.295(−17.936～−2.654)	0.009	−0.256(−0.541～0.028)	0.077
Maximum stone diameter (mm)	2.719 (1.868～3.570)	＜0.001	−0.004(−0.036～0.027)	0.794
Preoperative urine culture (positive)	−2.880(−10.843～5.082)	0.476	0.234(−0.062～0.531)	0.121
Stone location (kidney vs. ureter)	17.350 (4.428～30.273)	0.009	0.038(−0.444～0.519)	0.877

Reference group, modified prone split-leg position; B, unstandardized regression coefficient (a negative value indicates that the lithotomy position was lower than the modified prone split-leg position); CI, confidence interval; models for both outcomes were adjusted for maximum stone diameter, preoperative urine culture, and stone location.

In the multivariable logistic regression model for postoperative residual stones, the lithotomy position was associated with a significantly higher risk of residual stones compared with the modified prone split-leg position (OR = 5.327, 95% CI 1.426–19.904, *P* = 0.013). Maximum stone diameter was also independently associated with residual stones (OR = 1.179, 95% CI 1.062–1.310, *P* = 0.002). Preoperative urine culture positivity (OR = 0.873, 95% CI 0.276–2.759, *P* = 0.816) and stone location (OR = 2.165, 95% CI 0.545–8.595, *P* = 0.272) were not significantly associated with residual stones. Because the number of residual-stone events was limited (17 events), a sensitivity analysis was performed using Firth’s penalized logistic regression; the results were consistent with the primary analysis (lithotomy position: OR = 4.595, 95% CI 1.335–15.819, *P* = 0.016), supporting the robustness of the association.

In the multiple linear regression model for operative time, the lithotomy position was associated with a shorter operative time compared with the modified prone split-leg position (B = −10.295, 95% CI −17.936 to −2.654, *P* = 0.009), after adjusting for the same covariates. Maximum stone diameter (B = 2.719, 95% CI 1.868–3.570, *P* < 0.001) and stone location (B = 17.350, 95% CI 4.428–30.273, *P* = 0.009) were also independently associated with operative time. For postoperative hospital stay, no significant association was observed with surgical position (B = −0.256, 95% CI −0.541 to 0.028, *P* = 0.077).

### 3.4 Subgroup analyses

#### 3.4.1 Stratification by maximum stone diameter

In the subgroup with maximum stone diameter <10 mm, there were 31 patients in the observation group (modified prone split-leg position) and 39 patients in the control group (lithotomy position). The difference in postoperative day 1 stone-free rate between the two groups was not statistically significant (100% vs. 94.87%, *P* = 0.499) (Table 6). No statistically significant differences were observed between the two groups in operative time (*P* = 0.287), postoperative hospital stay (*P* = 0.197), change in plasma creatinine (*P* = 0.435), change in plasma urea nitrogen (*P* = 0.100), change in C-reactive protein (*P* = 0.334), or change in white blood cell count (*P* = 0.975).

**Table 6:** Comparison of surgical outcomes between the two groups for stones <10 mm.

Variable	Observation group (*n* = 31)	Control group (*n* = 39)	t/X^2^/Z/OR	*p*
Postoperative day 1 SFR, *n* (%)	31 (100%)	37 (94.87%)	—	0.499
Operative time (min), median (P25, P75)	35.00 (25.00,55.00)	31.00 (25.00,46.00)	−1.065	0.287
Postoperative hospital stay (d), median (P25, P75)	1.00 (1.00,2.00)	1.00 (1.00,1.00)	−1.290	0.197
Change in plasma urea nitrogen (mmol/L), median (P25, P75)	−0.62(−1.17,0.40)	−0.94(−1.68,−0.32)	−1.644	0.100
Change in plasma creatinine (μmol/L), median (P25, P75)	0.40(−5.00,5.60)	−1.10(−10.90,4.20)	−0.780	0.435
Change in C-reactive protein (mg/L), median (P25, P75)	0.00(−0.00,0.71)	0.00 (0.00,8.05)	−0.967	0.334
Change in white blood cell count (×10⁹/L), mean ± SD	1.04 ± 2.36	1.02 ± 3.22	0.032	0.975

In the subgroup with maximum stone diameter of 10–20 mm, there were 41 patients in the observation group (modified prone split-leg position) and 41 patients in the control group (lithotomy position). The postoperative day 1 stone-free rate was significantly higher in the observation group than in the control group (95.12% vs. 78.05%, *P* = 0.023). Operative time was longer in the observation group than in the control group (55 vs. 42 min, *P* = 0.005), and postoperative hospital stay was also longer (*P* = 0.023). No statistically significant differences were observed between the two groups in the change in plasma creatinine (*P* = 0.660), change in plasma urea nitrogen (*P* = 0.461), change in C-reactive protein (*P* = 0.341), or change in white blood cell count (*P* = 0.104) (Table 7).

**Table 7:** Comparison of surgical outcomes between the two groups for stones with maximum diameter 10–20 mm.

Variable	Observation group (*n* = 41)	Control group (*n* = 41)	t/X^2^/Z/OR	*p*
Postoperative day 1 SFR, *n* (%)	39 (95.12%)	32 (78.05%)	5.145	0.023
Operative time (min), median (P25, P75)	55 (45.50,68.50)	42.00 (32.50,59.00)	−2.780	0.005
Postoperative hospital stay (d), median (P25, P75)	1.00 (1.00,2.00)	1.00 (1.00,1.00)	−2.267	0.023
Change in plasma urea nitrogen (mmol/L), median (P25, P75)	−1.05(−2.01,−0.09)	−1.09(−2.05,−0.33)	−0.737	0.461
Change in plasma creatinine (μmol/L), median (P25, P75)	−2.40(−12.05, 3.80)	−2.90(−7.90,3.00)	−0.441	0.660
Change in C-reactive protein (mg/L), median (P25, P75)	0.00 (0.00,0.00)	0.00 (0.00,0.93)	−0.952	0.341
Change in white blood cell count (×10⁹/L), median (P25, P75)	0.08(−0.67,2.19)	0.95(−0.24,2.86)	−1.628	0.104

Given the small sample size of stones ≥20 mm (12 in the modified prone split-leg position and 4 in the lithotomy position), the statistical power was insufficient (<15%). Moreover, RIRS is not the mainstream indication for renal stones ≥20 mm. Therefore, this subgroup was not included in the formal subgroup analysis.

#### 3.4.2 Stratification by BMI

According to the Chinese Guidelines for the Prevention and Control of Overweight and Obesity in Adults, patients were divided into two groups: BMI <24 kg/m² (normal weight) and BMI ≥24 kg/m² (overweight/obese) for subgroup analysis.

In the subgroup with BMI <24 kg/m² (normal weight), there were 34 patients in the observation group (modified prone split-leg position) and 31 patients in the control group (lithotomy position). The difference in postoperative day 1 stone-free rate between the two groups was not statistically significant (94.12% vs. 83.87%, *P* = 0.352) (Table 8). Operative time was longer in the observation group than in the control group (50.00 vs. 40.00 min, *P* = 0.023), and postoperative hospital stay was also longer (*P* = 0.035). No statistically significant differences were observed between the two groups in the change in plasma creatinine (*P* = 0.115), change in plasma urea nitrogen (*P* = 0.296), change in C-reactive protein (*P* = 0.573), or change in white blood cell count (*P* = 0.391).

**Table 8:** Comparison of surgical outcomes between the two groups for BMI <24 kg/m².

Variable	Observation group (*n* = 34)	Control group (*n* = 31)	t/X^2^/Z/OR	*P*
Postoperative day 1 SFR, *n* (%)	32 (94.12%)	26 (83.87%)	0.866	0.352
Operative time (min), median (P25, P75)	50.00 (35.00,70.75)	40.00 (27.00,47.00)	−2.274	0.023
Postoperative hospital stay (d), median (P25, P75)	1.00 (1.00,2.00)	1.00 (1.00,1.00)	−2.114	0.035
Change in plasma urea nitrogen (mmol/L), mean ± SD	−1.04 ± 1.16	−1.42 ± 1.63	1.055	0.296
Change in plasma creatinine (μmol/L), median (P25, P75)	−0.10(−9.23,3.20)	−2.90(−11.40,0.00)	−1.576	0.115
Change in C-reactive protein (mg/L), median (P25, P75)	0.00 (0.00,0.00)	0.00 (0.00,0.00)	−0.564	0.573
Change in white blood cell count (×10⁹/L), mean ± SD	1.36 ± 2.24	0.76 ± 3.25	0.865	0.391

In the subgroup with BMI ≥24 kg/m² (overweight/obese), there were 50 patients in the observation group (modified prone split-leg position) and 53 patients in the control group (lithotomy position). The difference in postoperative day 1 stone-free rate between the two groups was not statistically significant (94.00% vs. 86.79%, *P* = 0.367). Operative time was longer in the observation group than in the control group (55.00 vs. 37.00 min, *P* = 0.002). No statistically significant differences were observed between the two groups in postoperative hospital stay (*P* = 0.156), change in plasma creatinine (*P* = 0.165), change in plasma urea nitrogen (*P* = 0.062), change in C-reactive protein (*P* = 0.643), or change in white blood cell count (*P* = 0.125) (Table 9).

**Table 9:** Comparison of surgical outcomes between the two groups for BMI ≥24 kg/m².

Variable	Observation group (*n* = 50)	Control group (*n* = 53)	t/X^2^/Z/OR	*P*
Postoperative day 1 SFR, *n* (%)	47 (94.00%)	46 (86.79%)	0.813	0.367
Operative time (min), median (P25, P75)	55.00 (38.75,72.50)	37.00 (29.50,57.50)	−3.027	0.002
Postoperative hospital stay (d), median (P25, P75)	1.00 (1.00,2.00)	1.00 (1.00,1.00)	−1.420	0.156
Change in plasma urea nitrogen (mmol/L), median (P25, P75)	−0.60(−1.31,0.21)	−0.91(−1.85,−0.29)	−1.867	0.062
Change in plasma creatinine (μmol/L), median (P25, P75)	0.75(−5.88,7.83)	−1.10(−7.60,4.00)	−1.389	0.165
Change in C-reactive protein (mg/L), median (P25, P75)	0.00 (0.00,0.05)	0.00(−0.05,0.00)	−0.463	0.643
Change in white blood cell count (×10⁹/L), median (P25, P75)	0.60(−0.59,1.91)	1.10 (0.12,2.79)	−1.534	0.125

#### 3.4.3 Stratification by stone location

In the renal stone subgroup, there were 13 patients in the observation group and 7 in the control group. The postoperative day 1 stone-free rate was significantly higher in the observation group than in the control group (92.31% vs. 42.86%, *P* = 0.031, OR = 10.71). Operative time was longer in the observation group than in the control group (104.15 ± 37.74 vs. 47.29 ± 19.65 min, *P* = 0.002). No statistically significant differences were observed between the two groups in postoperative hospital stay (*P* = 0.363), change in plasma creatinine (*P* = 0.153), change in plasma urea nitrogen (*P* = 0.313), change in C-reactive protein (*P* = 1.000), or change in white blood cell count (*P* = 0.322). However, the sample size of this subgroup was small (*n* = 20), resulting in insufficient statistical power (<70%), and the 95% confidence interval for the OR was extremely wide (1.19–96.25). Therefore, the above findings should be considered exploratory, suggesting that the modified prone split-leg position may have a stone-clearance advantage in RIRS for renal stones, but no definitive conclusion can be drawn, and large-sample prospective studies are needed for validation (Table 10).

**Table 10:** Comparison of surgical outcomes between the two groups for renal stones.

Variable	Observation group (*n* = 13)	Control group (*n* = 7)	t/X^2^/Z/OR	*P*
Postoperative day 1 SFR, *n* (%)	12 (92.31%)	3 (42.86%)	10.71 (1.19∼96.25)	0.031
Operative time (min), mean ± SD	104.15 ± 37.74	47.29 ± 19.65	3.694	0.002
Postoperative hospital stay (d), median (P25, P75)	1.00 (1.00,2.00)	1.00 (1.00,2.00)	−0.910	0.363
Change in plasma urea nitrogen (mmol/L), mean ± SD	−0.67 ± 1.07	−1.15 ± 0.93	1.046	0.313
Change in plasma creatinine (μmol/L), mean ± SD	4.42 ± 11.43	−1.19 ± 5.34	1.492	0.153
Change in C-reactive protein (mg/L), median (P25, P75)	0.00 (0.00,0.00)	0.00 (0.00,0.00)	0.000	1.000
Change in white blood cell count (×10⁹/L), median (P25, P75)	1.25 (0.47,3.72)	0.11(−0.47,2.08)	−0.991	0.322

In the ureteral stone subgroup, there were 71 patients in the observation group and 77 in the control group. The postoperative day 1 stone-free rates were 94.37% and 89.61%, respectively, with no statistically significant difference (*P* = 0.290). Operative time was longer in the observation group than in the control group [50.00 (35.00, 60.00) min vs. 38.00 (28.00, 50.00) min, *P* = 0.006]. Postoperative hospital stay was also longer in the observation group [1 (1, 2) d vs. 1 (1, 1) d, *P* = 0.024]. No statistically significant differences were observed between the two groups in the change in plasma creatinine (*P* = 0.193), change in plasma urea nitrogen (*P* = 0.223), change in C-reactive protein (*P* = 0.954), or change in white blood cell count (*P* = 0.282) (Table 11).

**Table 11:** Comparison of surgical outcomes between the two groups for ureteral stones.

Variable	Observation group (*n* = 71)	Control group (*n* = 77)	t/X^2^/Z/OR	*P*
Postoperative day 1 SFR, *n* (%)	67 (94.37%)	69 (89.61%)	1.121	0.290
Operative time (min), median (P25, P75)	50.00 (35.00,60.00)	38.00 (28.00,50.00)	−2.742	0.006
Postoperative hospital stay (d), median (P25, P75)	1.00 (1.00,2.00)	1.00 (1.00,1.00)	−2.257	0.024
Change in plasma urea nitrogen (mmol/L), mean ± SD	−0.88 ± 1.45	−1.17 ± 1.40	1.225	0.223
Change in plasma creatinine (μmol/L), median (P25, P75)	−0.20(−9.90,5.30)	−2.90(−9.60,3.55)	−1.303	0.193
Change in C-reactive protein (mg/L), median (P25, P75)	0.00 (0.00,0.00)	0.00 (0.00,0.00)	−0.058	0.954
Change in white blood cell count ( × 10⁹/L), median (P25, P75)	0.46(−0.51,2.46)	1.10(−0.49,2.79)	−1.077	0.282

#### 3.4.4 Interaction analysis

To assess whether the effect of surgical position on postoperative residual stones differed across subgroups, interaction analyses were performed for stone size, BMI, and stone location (Table 12). The *P for interaction* was 0.999 for stone size, 0.946 for BMI, and 0.059 for stone location. These findings indicate that the differential effect of surgical position across subgroups was not statistically confirmed. In the renal stone subgroup, the OR was extremely high with a very wide confidence interval, reflecting the very small sample size (*n* = 20) and unstable estimate.

**Table 12:** Logistic regression analysis of surgical position and postoperative residual stones across subgroups (*n* = 168).

Subgroup	*n*	OR (95%CI)	*P*	*P* for interaction
Maximum stone diameter (mm)	152			0.999
＜10	70	Not estimable	0.499	
10–20	82	6.58 (1.25～34.7)	0.027	
BMI (kg/m²)	168			0.946
＜24	65	10.60 (0.97～116.2)	0.053	
≥24	103	5.23 (0.75～36.4)	0.065	
Stone location	168			0.059
Ureteral	148	2.43 (0.63～9.42)	0.199	
Renal	20	125.05 (1.85～8433.4)	0.025	

1. After adjustment for maximum stone diameter and preoperative urine culture, OR for the lithotomy position versus the modified prone split-leg position (dependent variable: 1 = residual; reference group = modified prone split-leg position). 2. Wald test *P* value for the “surgical position × subgroup” interaction term in the logistic model. 3. The stone size subgroup analysis excluded stones ≥20 mm (*n* = 16). 4. In the <10 mm subgroup, no residual stone events occurred in the modified prone split-leg position group, so the OR could not be estimated, and the *P* value was derived from Fisher’s exact test.

## 4 Discussion

Upper urinary tract calculi are a common urological disease that can cause renal colic, urinary tract obstruction, and renal function impairment. As the mainstream minimally invasive treatment, the choice of intraoperative position during RIRS has an important impact on surgical outcomes (4).

In this study, propensity score matching was used to compare the clinical outcomes of the modified prone split-leg position versus the lithotomy position in RIRS for upper urinary tract calculi. The results showed that the immediate radiographic stone-free rates on postoperative day 1 were 94.05% and 85.71% in the two groups, respectively, with no statistically significant difference (*P* = 0.073). However, after adjustment for residual confounders, the lithotomy position was independently associated with a higher risk of residual stones compared with the modified prone split-leg position (OR = 5.327, 95% CI 1.426–19.904, *P* = 0.013), suggesting that the modified prone split-leg position may be associated with better stone clearance after controlling for confounding factors. Other studies have shown that the modified prone split-leg position can achieve a single-session stone-free rate of 78.1% in combined endoscopic surgery for multiple stones, with complications mainly grade 1–2, further confirming the safety of this position in complex cases (8).

In the present study, operative time was significantly longer in the modified prone split-leg position (54.00 vs. 38.50 min, *P* < 0.001), but there were no significant differences in postoperative fever rate, inflammatory markers, or renal function between the two groups. However, because complications were not systematically recorded using a standardized classification such as Clavien-Dindo, these findings should be interpreted as preliminary safety indicators rather than definitive evidence of safety. Subgroup analysis showed that the immediate radiographic stone-free rate was significantly higher in the observation group for stones of 10–20 mm (95.12% vs. 78.05%, *P* = 0.023); the renal stone subgroup also showed a higher stone-free rate (92.31% vs. 42.86%, *P* = 0.031), but due to the small sample size, this finding is only exploratory; the ureteral stone subgroup showed no significant difference. These results suggest that the modified prone split-leg position did not differ significantly from the lithotomy position in overall efficacy and may have an advantage for medium-sized stones.

Notably, the modified prone split-leg position has unique advantages that the lithotomy position cannot match. First, this position facilitates intraoperative conversion of the surgical approach. For patients with anticipated difficulty in lithotripsy or a risk of intraoperative conversion to percutaneous nephrolithotomy (PCNL), the modified prone split-leg position allows direct PCNL without repositioning, avoiding the time delay and contamination risk caused by changing the position. The study by Wang et al. showed that the modified prone split-leg position was designed to accommodate both RIRS and PCNL, facilitating combined endoscopic surgery (8). Second, this position facilitates intraoperative ultrasound examination. In the modified prone split-leg position, the patient’s lower back and abdomen are fully exposed, allowing the surgeon to use ultrasound in real time to assess stone location, fragmentation, and residual stones, thereby improving the thoroughness of stone clearance and reducing the postoperative residual stone rate. In contrast, in the lithotomy position, the patient’s lower back is obscured by the operating table, limiting ultrasound examination and making it difficult to assess stone clearance intraoperatively. A study by Cao et al. also confirmed that in the split-leg prone position, visual puncture PCNL is feasible, and intraoperative ultrasound guidance helps improve stone clearance accuracy (20).

The immediate radiographic stone-free rates in both groups in this study were within the range of KUB-assessed rates reported in the literature for RIRS (85%–95%) (2, 21). Although the absolute stone-free rate was about 8.34% higher in the observation group, the difference did not reach statistical significance, which may be related to the limited sample size and possible beta error. Previous comparative studies on RIRS positions have yielded inconsistent conclusions: some studies have suggested that the prone position or the split-leg prone position can increase the stone-free rate for lower-calyceal stones (7, 8), while others have found no significant difference (4). After controlling for confounding bias, the present study found that the modified prone split-leg position did not demonstrate superiority in the overall population, and no statistically significant difference was observed between the two groups, providing a reference for clinical positioning decisions.

In the multivariable logistic regression analysis adjusting for residual confounders, the lithotomy position remained independently associated with a higher risk of residual stones compared with the modified prone split-leg position (OR = 5.327, 95% CI 1.426–19.904, *P* = 0.013). This finding is consistent with the subgroup analysis and supports the potential advantage of the modified prone split-leg position in stone clearance. However, the interaction analyses showed that the *P for interaction* was 0.999 for stone size, 0.946 for BMI, and 0.059 for stone location, indicating that the differential effect of position across subgroups was not statistically confirmed. Therefore, the subgroup findings should be interpreted as exploratory.

The operative time with the modified prone split-leg position was prolonged by approximately 15.5 min compared with the lithotomy position. The prolonged operative time may be partly attributable to the complexity of patient positioning and to a possible learning curve. However, because surgeon case sequence and individual learning curves were not recorded in this retrospective dataset, this remains speculative and cannot be definitively distinguished from other factors. Additional exploration time may also be required because of stone migration caused by gravity (22). Although operative time was prolonged, the postoperative fever rate in both groups was 1.19%, which is lower than reported levels in the literature (14, 23), and there were no significant differences in inflammatory markers such as C-reactive protein and white blood cell count, suggesting that, within the limits of the available safety indicators, the prolonged operative time was not associated with an increased risk of infection or systemic inflammatory response. The median postoperative hospital stay was 1 day in both groups, and although the difference was statistically significant, its clinical value was limited.

Postoperative plasma urea nitrogen was higher in the observation group than in the control group (*P* = 0.013), but there were no statistically significant differences in plasma creatinine or in the changes of any of the indicators. Plasma urea nitrogen is easily influenced by non-renal factors such as dehydration, diet, and protein catabolism (24), so the clinical significance of this difference should be interpreted with caution. Previous studies have confirmed that renal function parameters (including plasma creatinine and plasma urea nitrogen) remain stable or improve after RIRS, indicating that the procedure has a minor impact on renal function (16). There were no significant differences in the changes of C-reactive protein or white blood cell count between the two groups, further supporting that the modified prone split-leg position does not increase systemic inflammation or renal injury.

The subgroup of stones 10–20 mm in maximum diameter was the most important positive finding of this study. In this subgroup, the immediate radiographic stone-free rate in the observation group was significantly higher than that in the control group (95.12% vs. 78.05%, *P* = 0.023). The mechanism may be that for medium-sized stones, the modified prone split-leg position uses gravity to concentrate stone fragments into the renal pelvis or upper calyx (22), facilitating thorough exploration and laser lithotripsy; at the same time, this position may improve stone exposure through gravity assistance and reduce stone fragment migration into less accessible calyces (25). In addition, the split-leg design facilitates transurethral manipulation, allowing the surgeon to flexibly adjust the angle of the flexible ureteroscope (8). These findings suggest that the modified prone split-leg position may be associated with a higher SFR in patients with stones of 10–20 mm. However, because the interaction between surgical position and stone size was not statistically significant (*P for interaction* = 0.999), this result should be interpreted as exploratory and requires confirmation in adequately powered studies.

In the renal stone subgroup, the modified prone split-leg position showed a numerically higher stone-free rate than the lithotomy position (92.31% vs. 42.86%, *P* = 0.031, OR = 10.71). However, this subgroup had only 20 patients (13 in the observation group and 7 in the control group), with insufficient statistical power (<70%), and the 95% confidence interval for the OR was extremely wide (1.19–96.25), making the result very unstable. Therefore, this finding should be considered hypothesis-generating and cannot lead to a definitive conclusion. Previous studies have reported that the prone position has anatomical advantages in the management of lower-calyceal stones (9), and the present results are consistent with that, but large-sample prospective studies are needed for validation. We suggest that future studies conduct adequately powered randomized controlled trials for renal stones (especially lower-calyceal stones).

In the ureteral stone subgroup, there was no significant difference in stone-free rate between the two groups (94.37% vs. 89.61%, *P* = 0.290), but operative time and hospital stay were longer in the observation group. Ureteral stones are relatively fixed and do not move with changes in body position, so the advantage of position optimization for stone clearance is not obvious. This result suggests that the advantage of the modified prone split-leg position may be mainly in renal stones, while for simple ureteral stones, the lithotomy position remains a reasonable choice, avoiding unnecessary time for positioning.

In the subgroups of maximum stone diameter <10 mm and different BMI categories, there were no significant differences in stone-free rates. Small-sized stones are easily cleared regardless of the position, so the choice of position has little effect on the stone-free rate (26). BMI did not affect the efficacy of the position, suggesting that the modified prone split-leg position is applicable to patients of different body types, which may be attributable to the good adaptability of the split-leg design for obese patients.

This study has the following limitations: A major limitation is that surgical position was completely confounded with study site: the modified prone split-leg position was routinely used at one campus and the lithotomy position at the other. Therefore, the observed associations may partly reflect between-site differences in equipment configuration, operating-room workflow, or ancillary staff, rather than the position itself. Our findings should thus be interpreted as associations rather than causal effects of body position. Although propensity score matching was used to control for observable confounding, the retrospective design cannot completely eliminate the influence of unmeasured confounding factors (27). In addition, although most covariates were well balanced after PSM, the SMDs for maximum stone diameter (0.317) and preoperative urine culture positivity (0.297) slightly exceeded 0.25, indicating residual imbalance. Multivariable regression was therefore performed to further control for these confounders. Although the association remained significant in the penalized sensitivity analysis, the limited number of events means that the confidence interval remains wide, and the adjusted association should be interpreted with appropriate caution. Furthermore, the interaction analyses were not statistically significant, and the subgroup findings should be interpreted as exploratory. The small sample size of the renal stone subgroup may lead to beta error; subgroup analyses were not adjusted for multiple comparisons, posing a risk of false positives; the stone-free rate was assessed only by KUB on postoperative day 1. KUB is less sensitive than CT for detecting radiolucent stones and small residual fragments, which may overestimate the true SFR. Although some patients underwent subsequent CT evaluation based on clinical needs, CT was not performed in all patients, and these data were not systematically analyzed. Therefore, no CT-adjusted SFR analysis was performed, and the lack of 3-month CT follow-up may overestimate the immediate stone-free rate (28); the single-institution data and the lack of recording of intrarenal pressure, patient comfort, and other indicators also limit the generalizability and comprehensiveness of the conclusions. Other perioperative complications were not systematically recorded using a standardized classification such as Clavien-Dindo. In addition, a formal learning curve analysis was not feasible because surgeon case sequence was not recorded. Moreover, because only endoscopic procedure time was recorded, total operating-room time and patient positioning time could not be compared between the two positions; these outcomes are clinically relevant when comparing surgical positions and should be collected in future studies.

Based on the findings of this study, future research can be explored in the following directions: (a) conduct multicenter, prospective randomized controlled trials with the stone-free rate assessed by 3-month CT as the primary endpoint to validate the conclusions of this study; (b) perform stratified randomization for patients with stones of 10–20 mm and lower-calyceal stones to clarify the optimal indications for the modified prone split-leg position; (c) explore the learning curve for operative time and positioning time with the modified prone split-leg position to assess the impact of surgeon proficiency on efficacy and operative time; (d) include patient-reported outcome measures (such as postoperative comfort, recovery quality, and position-related discomfort) to comprehensively evaluate the advantages and disadvantages of the two positions.

In summary, the modified prone split-leg position in RIRS showed an immediate radiographic stone-clearing effect that did not differ significantly from that of the lithotomy position, with no apparent short-term safety concerns based on the available indicators. For upper urinary tract stones of 10–20 mm, the modified prone split-leg position may improve the immediate stone-free rate, which has certain clinical value. Due to the retrospective design and sample size limitations in some subgroups, the above conclusions need to be further validated by prospective, large-sample studies. This study provides new evidence-based support for the choice of position during RIRS.

## 5 Conclusion

In this retrospective propensity score-matched study, the modified prone split-leg position in RIRS for upper urinary tract calculi showed no statistically significant difference in the immediate radiographic stone-free rate compared with the lithotomy position in the unadjusted analysis, with no apparent short-term safety concerns based on the available indicators. However, after adjustment for residual confounders, the lithotomy position was independently associated with a higher risk of residual stones, suggesting a potential advantage of the modified prone split-leg position for stone clearance, although this association may partly reflect between-site differences rather than the effect of position *per se*. A higher stone-free rate was observed for stones of 10–20 mm; however, the interaction was not statistically significant, and this subgroup finding should be interpreted cautiously and requires validation in larger studies. In the renal stone subgroup, a numerically higher SFR was observed, but this finding is hypothesis-generating because of the small sample size and wide confidence interval, and it requires validation by large-sample prospective studies.

## Data Availability

The original contributions presented in the study are included in the article/Supplementary Material, further inquiries can be directed to the corresponding author/s.
